# Fat mass and obesity-associated (*FTO*) gene epigenetic modifications in gestational diabetes: new insights and possible pathophysiological connections

**DOI:** 10.1007/s00592-020-01668-5

**Published:** 2021-03-20

**Authors:** Marica Franzago, Federica Fraticelli, Michele Marchioni, Marta Di Nicola, Francesca Di Sebastiano, Marco Liberati, Liborio Stuppia, Ester Vitacolonna

**Affiliations:** 1 Department of Medicine and Aging, School of Medicine and Health Sciences, “G. D’Annunzio” University, Chieti-Pescara, Via dei Vestini, 66100 Chieti, Italy; 2 Center for Advanced Studies and Technology (CAST), “G. D’Annunzio” University, Chieti-Pescara, Chieti, Italy; 3 Laboratory of Biostatistics, Department of Medical, Oral and Biotechnological Sciences, “G.D’Annunzio” University, Chieti-Pescara, Chieti, Italy; 4 Department of Obstetric and Gynaecology, SS. Annunziata Hospital, “G. D’Annunzio” University, Chieti-Pescara, Chieti, Italy; 5 Department of Psychological, Health and Territorial Sciences, School of Medicine and Health Sciences, “G. D’Annunzio” University, Chieti-Pescara, Chieti, Italy

**Keywords:** Gestational diabetes, DNA methylation, Epigenetics, Obesity, *FTO*, Maternal smoke, rs9939609

## Abstract

**Aims:**

Gestational diabetes mellitus (GDM) can lead to short- and long-term complications for the child. Epigenetic alterations could contribute to explaining the metabolic disturbances associated with foetal programming. Although the role of the *FTO* gene remains unclear, it affects metabolic phenotypes probably mediated by epigenetic mechanisms. The aim of this study was to assess whether placental DNA epigenetic modifications at *FTO* promoter-associated cysteine–phosphate–guanine (CpG) sites are correlated with GDM. A secondary aim was to evaluate the association between the placental *FTO* DNA methylation and the maternal metabolic traits in women with and without GDM.

**Methods:**

Socio-demographic characteristics, clinical parameters at the third trimester of pregnancy, Mediterranean diet adherence, and physical activity were assessed in 33 GDM women and 27 controls. Clinical information about the newborns was registered at birth. The *FTO* rs9939609 (T > A) was genotyped.

**Results:**

No association between *FTO* DNA methylation and GDM was found. DNA methylation on the maternal side at the CpG1 was associated with maternal smoking in GDM (*p* = 0.034), and DNA methylation at the CpG3 was correlated with smoking or former smoking in controls (*p* = 0.023). A higher level of TGs was correlated with higher foetal placental DNA methylation at the CpG2 (*p* = 0.036) in GDM. An inverse association between HDL-C and maternal placental DNA methylation at the CpG3 in controls (*p* = 0.045) was found. An association between *FTO* rs9939609 and neonatal birthweight (*p* = 0.033) was detected.

**Conclusions:**

In the awareness that the obesity pathophysiology is complex, the study adds a piece to this intricate mosaic.

**Supplementary Information:**

The online version of this article (10.1007/s00592-020-01668-5) contains supplementary material, which is available to authorized users.

## Introduction

GDM is the most common metabolic disorder, and it is defined as diabetes diagnosed in the second or third trimester of pregnancy that was not clearly overt diabetes prior gestation [1]. GDM is associated with an increased risk of future health complications for mother and child, including adverse perinatal outcomes, high subsequent risk of type 2 diabetes (T2DM), metabolic syndrome, and cardiovascular disease [2–5]. Multiple risk factors for GDM have already been identified, including increased body mass index (BMI) and weight gain during pregnancy, ethnicity, family history of diabetes, advanced maternal age, and sedentary lifestyle before or during pregnancy. Growing evidence suggests that also the complex interaction between genetics, epigenetics, and diverse environmental factors plays a role in the pathogenesis of GDM [6–11].

Some maternal metabolic factors, including pre-pregnancy overweight/obesity and GDM, influence the foetal growth trajectories and affect children’s susceptibility to lifetime chronic diseases, including possible transgenerational effects [12, 13]. This mechanism fits well with the Developmental Origins of Health and Disease (DOHaD) hypothesis, which proposes that the origin of chronic diseases is related to a prenatal exposure to a suboptimal foetal environment [14, 15]. In this view, the exact mechanism responsible for the complexity of metabolic status in GDM is poorly understood, but some studies have observed epigenetic dysregulation in foetal metabolic programming of newborns exposed to maternal hyperglycaemia during pregnancy. Several authors have investigated the potential associations between DNA methylation in placenta collected at birth and maternal hyperglycaemia, since the former is a critical protagonist in the regulation of foetal growth and development. In its role as a controller of maternal foetal nutrient exchanges via epigenetic mechanisms, placenta responds to foetal demands and maternal availability of nutrients [16].

To date, the vast majority of studies have focused on DNA methylation changes in candidate genes mainly involved in the pathways of energy balance, glucose and lipid metabolism, and their association with GDM [17–21].

At present, the correlation between the methylation profiles of fat mass and obesity-associated (*FTO*) gene on the maternal and foetal sides of the placenta and GDM remains unexplored. The *FTO* gene encodes an RNA demethylase which is expressed predominantly in the hypothalamus as well as across other tissues, including placenta. Increased FTO mRNA levels are associated with higher foetal weight and length as well as higher placental weight, suggesting *FTO* as a regulator of the partitioning between placenta and foetal growth [22].

Single nucleotide polymorphisms (SNPs) in *FTO* are the strongest known genetic risk factors for obesity and have been linked with BMI, GDM, T2DM, and eating behaviour [8, 23–26]. Although the functions of *FTO* remain undefined, some studies demonstrated that it influences the expression of IRX3, a homoeobox gene involved in pattern formation in the early embryo with effects on body weight [27, 28].

In addition, it has been suggested that *FTO* could be involved in the cellular sensing of amino acids, in the regulation of cell growth, and in the global mRNA translation through the mTORC1 pathway [29]. Placental amino acid transporters control foetal growth and regulate the supply of nutrients to the foetus. In this view, *FTO* could potentially regulate foetal growth by altering the placental amino acid transport [30]. To date, the exact mechanisms through which *FTO* gene influences growth and body composition are unknown.

The study of the epigenetic differences between placental tissues in GDM and in controls may contribute to explain higher long-term metabolic disturbances and obesity in GDM mothers and their newborns. Therefore, we hypothesize that maternal metabolic status could affect placenta’s *FTO* DNA methylation profile, influencing foetal metabolic programming. The present study aimed to evaluate the relationship between the exposure to an altered intrauterine environment and foetal metabolic programming, focusing on GDM and DNA methylation profiles at promoter-associated CpG islands of the *FTO* gene.

## Materials and methods

### Study design and participants

Sixty Caucasian pregnant women attending the Diabetes, Nutrition and Metabolism Unit and the Obstetrics and Gynaecology Clinic, School of Medicine and Health Sciences, “G. D’Annunzio” University of Chieti-Hospital “SS Annunziata” of Chieti, were recruited.

The study was approved by the Ethics Committee of the “G. D’Annunzio” University, Chieti-Pescara (Italy). In compliance with the Declaration of Helsinki, all subjects provided a written informed consent before their inclusion in the study.

During the first visit, data on demographic characteristics were collected. Anthropometric parameters were measured and recorded according to standard procedures. Clinical parameters (blood glucose, lipid profile [total cholesterol (TC), high-density lipoprotein cholesterol (HDL-C), low-density lipoprotein cholesterol (LDL-C), triglycerides (TG)], and blood pressure) were recorded at the third trimester. Physical activity was assessed using a short version of the International Physical Activity Questionnaire (IPAQ), registering three different levels of intensity (low, moderate, and high PA) [31]. Adherence to the Mediterranean diet (MedDiet) was evaluated through a validated 14-item questionnaire (PREDIMED), which generates a range of possible scores, namely (i) no adherence (score ≤ 5), (ii) medium adherence (6 ≤ score ≤ 9), and (iii) maximum adherence (score ≥ 10) [32].

At delivery, placenta biopsies were collected. Clinical information about the newborns (including mode of delivery, gestational age, sex, and anthropometric measurements) was collected at birth.

### Inclusion and exclusion criteria

The inclusion criteria accepted women with ≥ 18 years of age. The GDM diagnosis was performed following the International Association of Diabetes and Pregnancy Study Groups (IADPSG) criteria [33, 34]. The exclusion criteria were type 1 or 2 diabetes, overt diabetes, other chronic diseases (including malignancy, hypercholesterolemia) as well as a positive history of drug or alcohol abuses.

### Placenta tissue sampling

Placenta tissues were sampled within a few minutes after delivery and placental expulsion. Two biopsies of 0.5 cm^3^ were taken on the foetal and maternal sides.

The procedures we deployed for placental tissue collection consisted in washing the tissue in order to remove the blood present in the original sample. This is an optimized procedure for epigenomic analysis in the clinical setting, as reported in the literature [18, 21, 35–38].

In detail, placental biopsies were washed in PBS 1 × to remove cord/maternal blood and dissected to remove conjunctive tissues. The samples were kept in RNAlater Stabilization Solution (Thermo Fischer Scientific, Waltham, MA, USA) at −80 °C until nucleic acid extraction. DNA was purified from placenta biopsies using MagPurix 12s Automated Nucleic Acid Purification System (Zinexts Life Science Corp., Taiwan) according to the manufacturer’s instructions.

### Epigenetic analysis (DNA methylation analysis)

DNA methylation levels at CpG sites were assessed using pyrosequencing (Pyromark Q96; Qiagen). In brief, genomic DNA was treated with sodium bisulphite (NaBis) using the BisulFlash DNA modification kit (EpiGentek), converting unmethylated cytosines to uracils. After bisulphite treatment, DNA (~ 20 ng) was amplified by PCR using the Kapa Hifi Hotstart Uracil + HotStart ReadyMix (Roche Diagnostics) and pyrosequenced according to manufacturer’s recommendations. The conditions in the PCR stage were 95 °C for 5 min, 40 cycles at 98 °C for 15 s, 60 °C for 30 s, and 72 °C for 7 min, and a final extension at 72 °C for 7 min.

The PCR and pyrosequencing primers for all four CpGs tested within the *FTO* gene were *FTO*F: 5′-TTTGGAGTTATTTTTTTTTTGAGTAGAAA-3′, *FTO*R: 5′-[Btn] ATTCTCCTTAAACTCTAACCTATTTACT-3′ (168 bp), and *FTO*Seq: 5′-TTTTAGGTTAGATAGTTGGAAGA-3′ (4 CpGs) according to previous studies [39].

Four of all DNA samples (two from the mother’s side and two from the foetal’s side) did not amplify and therefore were not analysed.

The specificity of pyrosequencing assays is 100% and the sensitivity is approximately 5%, in terms of percentage of methylated cytosines detectable in a sample containing both methylated and unmethylated cytosines [40, 41]. Nevertheless, the detection limit (LOD) for several mutant alleles (such as in *EGFR* and *KRAS* mutations) evaluated in routine diagnostic setting and supported by commercially available assays (Pyro Kit Qiagen) reaches up to 0.6%. Consequently, this supports the reliability to consider the LOD up to 1%.

### Genotyping

The rs9939609 (T > A) SNP in *FTO* was genotyped in all 60 pregnant women. All DNA samples were amplified by polymerase chain reaction (PCR) performed in 30-μl reaction volume containing 30 ng of genomic DNA in a SimpliAmp™ thermal cycler (Applied Biosystems™), using the AmpliTaq Gold DNA Polymerase. PCR conditions were as follows: initial denaturation at 95 °C for 10 min, followed by 35 cycles of 95 °C for 30 s, 60 °C for 30 s, 72 °C for 30 s, and a final extension at 72 °C for 10 min. The amplification products were submitted to direct sequencing procedure using BigDye Term v3.1 CycleSeq Kit (Life Technologies, Monza, Italy) followed by automatic sequencing analysis.

The specificity for Sanger sequencing is > 99% and the sensitivity is described as 20% mutated alleles in a background of wild-type alleles [40–43].

### Statistical analysis

Descriptive statistics relied on median and interquartile ranges (IQR) for quantitative variables and on absolute and relative frequencies for qualitative variables. The entire cohort was divided according to the GDM status. Differences in median were tested using the Mann–Whitney test, while differences in proportions were tested with the Chi-squared test, applying the Fisher’s exact correction when appropriated. Tobit models were fitted in order to test predictors of methylation levels among each island. We relied on tobit regression models that allow to account for left censored data, after observing the CpGs were mainly unmethylated. The tobit model assumed a normal distribution for the dependent variable with left-censoring at 0 [44].

A further stratification for both GDM and normoglycaemic (normal glucose tolerance, NGT) women was used according to the DNA methylation level of the different CpGs defining as “methylated” those women who showed at least 1% of DNA methylation for each CpG site or on the average. Concordance between methylated status for each CpG on the maternal and foetal side was also explored. Percentage of agreement was estimated, and the McNemar’s test was applied.

For the investigated SNP, Hardy–Weinberg equilibrium was calculated.

Given the exploratory nature of the current study, we did not adjust *p*-values to control family-wise error rate (FWER).

All the statistical analyses were performed using R Statistical Software (version 4.0.0, R Foundation for Statistical Computing, Vienna, Austria). All tests were two tailed, and a *p*-value < 0.05 was considered indicative of a statistically significant association.

## Results

### Demographic characteristics

Data from 60 mother–children couples were collected. The demographic and clinical characteristics of the women (GDM = 33 and NGT = 27) are reported in Table 1. Among women with GDM, insulin treatment became necessary for five subjects. The GDM diagnosis was performed at 16–18 weeks of gestation for six women and at 24–28 weeks for 26 women, respectively. GDM women showed a higher median BMI before pregnancy (25.2 vs. 21.6; *p* = 0.022) when compared with controls; an increase in weight gain at the delivery compared to the weight measured in pre-gestational period was observed in control group (10.0 vs. 12.0 kg; *p* = 0.037). Regarding lifestyle, we found no difference in the physical activity, MedDiet adherence, and smoking between the groups (Table 1). The main neonatal anthropometric characteristics of the study population are summarized in Table 2. No differences for gender distribution, birthweight, length, head circumferences, and APGAR scores were documented between the two groups.

**Table 1:** Clinical characteristics of healthy pregnant and GDM women

	NGT (*N* = 27)	GDM (*N* = 33)	*p-*Value
Age (year)	33.0 (29.0, 38.0)	35.0 (32.0, 38.0)	*0.114*^a^
Predimed, *n (%)*			*0.763*^b^
Maximum adherence	9 (33.3%)	8 (25.8%)	
Medium adherence	17 (63.0%)	21 (67.7%)	
No adherence	1 (3.7%)	2 (6.5%)	
IPAQ, *n* (%)			*0.237*^b^
Low	18 (66.7%)	17 (56.7%)	
Moderate	6 (22.2%)	12 (40.0%)	
High	3 (11.1%)	1 (3.3%)	
Smoking history, *n* (%)			*0.780*^b^
Non-smoker	17 (63.0%)	16 (59.3%)	
Smoker	10 (37.0%)	11 (40.7%)	
Pre-pregnancy weight (kg)	60.0 (53.5, 64.0)	67.0 (56.5, 85.0)	***0.008***^a^
Weight at the end of pregnancy (Kg)	71.0 (65.0, 77.5)	77.5 (68.0, 95.0)	***0.031***^a^
BMI at the end of pregnancy (Kg/m^2^)	27.0 (25.2, 27.9)	28.5 (25.1, 33.2)	*0.194*^a^
Pre-pregnancy BMI (Kg/m^2^)	21.6 (20.4, 23.3)	25.2 (21.2, 29.4)	***0.022***^a^
Weight variation (delivery vs pre-pregnancy) (Kg)	12.0 (10.0, 15.4)	10.0 (9.0, 12.5)	***0.037*** ^a^
Systolic blood pressure (mmHg)	110.0 (100.0, 120.0)	110.0 (107.5, 120.0)	*0.074* ^a^
Diastolic blood pressure (mmHg)	70.0 (60.0, 70.0)	75.0 (70.0, 80.0)	***0.003***^a^
HbA1c (%)	4.0 (4.0, 4.0)	5.2 (4.9, 5.3)	*0.093*^a^
Third-trimester TC (mg/dl)	256.5 (213.5, 282.5)	240.5 (231.2, 285.0)	*0.672* ^a^
Third-trimester HDL-C (mg/dl)	59.0 (53.5, 88.0)	68.5 (59.8, 82.0)	*0.740* ^a^
Third-trimester TG (mg/dl)	218.0 (187.5, 248.5)	195.0 (174.0, 252.2)	*0.666* ^a^
Third-trimester LDL-C (mg/dl)	241.9 (207.0, 260.0)	220.5 (208.5, 265.8)	*0.778* ^a^
OGTT (mg/dl) time 0	80.0 (75.0, 83.5)	92.0 (82.0, 95.0)	** < 0.001**^a^
OGTT (mg/dl) time 60 min	122.5 (111.5, 138.5)	168.5 (138.0, 187.2)	** < 0.001**^a^
OGTT (mg/dl) time 120 min	97.0 (84.8, 113.5)	139.5 (114.2, 152.2)	** < 0.001**^a^
First quarter fasting blood glucose (mg/dl)	80.0 (72.0, 83.8)	90.0 (80.8, 95.5)	*0.137*^a^

Data are expressed as median and interquartile range (IQR) for continuous variables and as frequencies and percentages for categorical variables

^a^Mann–Whitney *U* test

^b^Pearson’s Chi-squared test

**Table 2:** Neonatal outcomes relative to women with and without GDM

	NGT (*n* = 27)	GDM (*n* = 33)	*p*-Value
Gestational week	40.0 (39.0, 41.0)	39.0 (39.0, 40.0)	***0.002***^a^
Gender, *n(%)*			*0.802*^b^
Male	11 (42.3%)	11 (45.8%)	
Female	15 (57.7%)	13 (54.2%)	
Birthweight (grams)	3385.0 (3252.5, 3687.5)	3320.0 (3155.0, 3680.0)	*0.666*^a^
Birthweight (percentiles)	53.8 (38.9, 82.5)	63.6 (53.6, 83.3)	*0.209*^a^
One-minute Apgar scores	9.0 (9.0, 9.0)	9.0 (9.0, 9.0)	*0.504*^a^
Five-minute Apgar scores	10.0 (10.0, 10.0)	10.0 (10.0, 10.0)	*0.634*^a^
Birth head circumference (cm)	34.5 (34.0, 35.0)	35.0 (34.0, 36.0)	*0.448*^a^
Birth length (cm)	51.5 (50.0, 52.0)	50.0 (49.0, 51.0)	*0.138*^a^

Data are expressed as median and interquartile range (IQR) for continuous variables and as frequencies and percentages for categorical variables

^a^Mann–Whitney *U* test

^b^Pearson’s Chi-squared test

### DNA methylation analysis of the placental FTO gene

**Fig. 1:**
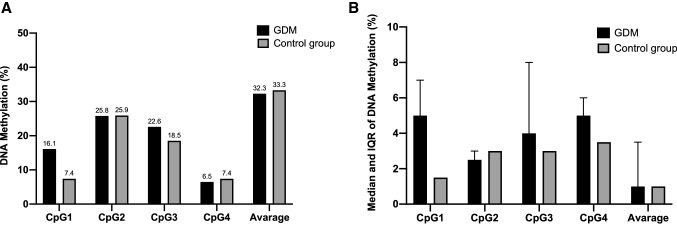
Percentage of women with at least 1% DNA methylation levels on the maternal side of placenta at the CpGs (Panel A). Median (IQR) of DNA methylation levels on the maternal side at the CpGs in women with at least 1% of DNA methylation (Panel B)

**Fig. 2:**
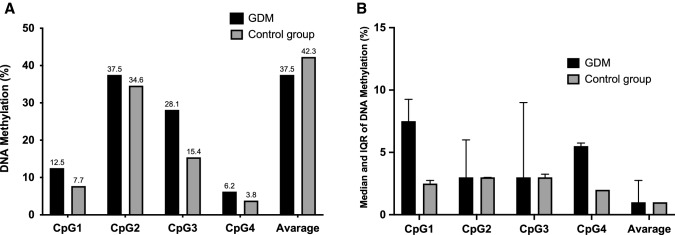
Percentage of women with at least 1% DNA methylation levels on the foetal side of placenta at the CpGs (Panel A). Median (IQR) of DNA methylation levels on the foetal side at the CpGs in patients with at least 1% of DNA methylation (Panel B)

The four CpGs tested within the promoter of *FTO* gene were mainly unmethylated in both groups. Among those who had at least 1% of methylation, the median of DNA methylation levels across all the examined islands on the maternal site of placenta was 1.0% (IQR 1–3.5%) and 1.0% (IQR 1.0–2.0%) for GDM and NGT, respectively (Fig. 1). Similarly, the median of DNA methylation levels on the foetal side of placenta was 1.0% (IQR 1.0–2.8%) for GDM and 1.0% (IQR 1.0–1.0%) for NGT, respectively (Fig. 2). Among women who had the DNA levels methylated, the GDM patients showed higher DNA methylation on the maternal as well as the foetal side at the CpG1, CpG3, and CpG4 when compared to the NGT group, although not statistically significant (Figs. 1 and 2).

The proportions of methylated islands at the foetal and maternal sides were similar in all the examined islands with high agreement confirmed by the McNemar’s test. In particular, in GDM patients the agreement was 90.0% (95% CI 73.5–97.9%) for CpG1, 70.0% (95% CI 50.6–85.3%) for CpG2, 83.3% (95% CI 65.3–94.4%) for CPG3, and 76.7% (95% CI 57.7–90.1%) for CpG4. Similarly, in NGT patients the agreement was 84.6% (95% CI 65.1–95.6%) for CpG1, 53.8% (95% CI 33.3–73.4%) for CpG-273.1% (95% CI 52.2–88.4%) for CpG3, and 88.5% (95% CI 69.9–97.6%) for CpG4.

Tobit models revealed no association among DNA methylation levels on both placenta sides and maternal clinical parameters, but it has been noted a trend towards significance between BMI before and at the end of pregnancy and DNA methylation on the maternal side at CpG1 site (*p* = 0.081 and 0.073, respectively) (Supplementary Table 1). In addition, there was a trend towards significance between DNA methylation on the foetal side and TGs (*p* = 0.070) and PAS (*p* = 0.092), respectively (Supplementary Table 2).

However, exploratory analyses investigating the association among GDM maternal clinical parameters and DNA methylation levels showed that the 66.7% of those who had at least 1% of DNA methylation on the maternal side at CpG1 were smoker vs. only the 9.1% of those who had DNA unmethylated levels (*p* = 0.034). Similarly, NGT women who presented DNA methylation levels on the maternal side at the CpG3 were more frequently smoker 20.0% or former smoker 60.0% than those with DNA unmethylated levels (smoker:0.0% and former smoker 27.3%, *p* = 0.023).

Among GDM women, no statistically significant differences in DNA methylation levels on both sides of the placenta were observed between women who performed OGTT early in pregnancy and those who did at 24–28 weeks of gestation (Supplementary Tables 3 and 4), but a trend towards significance between OGTT and DNA methylation on the maternal side at CpG1 site (p = 0.06) has been noted (data not shown).

**Table 3:** Genotypes distribution in GDM and healthy pregnant women

	GDM (*n* = 33)	No GDM (*n* = 27)	Chi-squared test *p*-value
*FTO* rs9939609(T > A)			*0.550*
*TT*	9 (27.3%)	5 (18.5%)	
*TA*	15 (45.5%)	16 (59.3%)	
*AA*	9 (27.3%)	6 (22.2%)	

Moreover, GDM women presenting DNA methylation levels on the foetal side at the CpG2 site had higher level of third-trimester maternal TGs (median 263.0 vs. 188.0 g/dl, *p* = 0.036). In addition, higher third-trimester maternal HDL-C has been found in NGT women with DNA unmethylated levels on maternal side at CpG3 site compared to those with DNA methylated levels (median 83.0 vs. 43.5, *p* = 0.045).

The genotypes distribution of rs9939609 (T > A) in GDM patients and in controls is reported in Table 3. No significant differences in genotype frequency were detected between GDM and controls. The genotype frequencies were within the Hardy–Weinberg equilibrium (χ^2^ *p* value > 0.05) both in cases and in controls. Interestingly, an association between *FTO* rs9939609 and neonatal birthweight expressed as percentiles was detected (*p* = 0.033).

## Discussion

Our study focused on methylation profiles of the *FTO* gene on both the maternal and foetal sides of the placenta in pregnant women with and without GDM.

Pregnancy can be considered a critical time of rapid physical and physiological changes. In this context, the placenta is a unique organ which influences maternal physiology and is likely to play a role in maternal insulin sensitivity changes during pregnancy. The placenta optimizes foetal growth, protects the foetus against infections, and produces key hormones to maintain pregnancy [16].

It has been shown that the offspring of women with aberrant alterations in metabolic pathways during pregnancy are at risk of developing future health complications [45–47], but the underlying mechanisms linking the maternal metabolic status to the offspring outcomes remain to be determined. DNA methylation, the most studied type of epigenetic mark occurring mostly at cytosine–phosphate–guanine dinucleotides, has been shown to be sensitive to environmental insults including in utero and postnatal environmental conditions [48–52]. In particular, data have shown that early-life exposures and their effects on lifetime metabolic disease are mediated by DNA methylation alterations in the gene pathways involved in the endocrine function, metabolism, and insulin responses.

Reichetzeder et al. [53] analysed the placental DNA methylation patterns of 1,030 pregnant women and revealed a significantly higher frequency of GDM in the group with the most methylated placental DNA. In this view, GDM is correlated with an altered placental function and changes in the placental gene regulation via epigenetic mechanisms [see review 4]. In particular, several studies have shown that GDM epigenetically affects genes that are predominantly involved in metabolic pathways [17, 19, 54].

*FTO* has been researched for a long time due to the strong association of its SNPs with energy homeostasis and body composition [55–57]. *FTO* influences the posttranscriptional regulation of gene expression promoting m6A demethylation on mRNA transcripts [58, 59], but its function is still largely unknown. A significant role of the hypothalamic *FTO* expression [60] as well as a relationship between *FTO* and weight gain [59, 61] due to several mRNA transcripts dynamically altered in response to diverse availabilities of energy and nutrient has been suggested [62, 63]. In addition, *FTO* overexpression leads to obesity and increased appetite [64], while loss protein evolves in lean phenotypes [65]. *FTO* is expressed and secreted by the placenta during pregnancy; also, several data suggest that *FTO* may regulate the transcription of genes involved in foetal growth by nucleic acid demethylation [22, 66].

In our study, we did not find any association between placental DNA methylation in the *FTO* gene in GDM women. At first, we found that CpG dinucleotides within *FTO* gene promoter were mainly unmethylated with only very limited DNA methylation level variability. We might speculate that the absence of correlation between GDM and DNA methylation levels in the *FTO* gene might be explained by a good maternal glycaemic control obtained by diet alone or diet and insulin from GDM diagnosis to delivery, thus alleviating the impact of GDM on the newborn’s epigenome. In support of this, as reported previously with regard to different analytic techniques, only modest changes on the placenta protein profile were observed in well-controlled GDM [67].

DNA methylation changes can be mediated by both genetic and modifiable lifestyle factors. In our sample, up to 40% of women smoked during pregnancy. Interestingly, noteworthy studies showed long-term effect on offspring adiposity induced by maternal smoke [68]. When considering exploratory analyses differentiating the DNA methylation levels in “unmethylated” and “methylated” in both groups, we found differences in the methylation patterns that occur in women exposed to tobacco smoke during pregnancy and that these differences were detected in placental DNA from the maternal side. The exposed GDM and NGT women had a significantly higher level of methylation at CpG1 and CpG3 site compared with unexposed ones. The association between tobacco smoking and altered DNA methylation patterns has been shown for a number of single genes, mostly related to inflammation, oxidative stress, and hypoxia, as well as in genome-wide methylation studies [69–71] showing some of the CpG sites associated with low birthweight in the offspring or decreased gestational age [72, 73]. So far, these studies have suggested that the maternal smoking deregulates the placental methylation in CpGs which correlates with alterations in gene expression along signature pathways [69, 74, 75].

It should be emphasised that the maternal conditions including lipids profile have been shown to be detrimental for foetal development and lipid metabolism independently of maternal hyperglycaemia [76, 77]. Interestingly, our findings showed a significant association between foetal placental DNA methylation levels and third-trimester TGs within the GDM group. Moreover, an inverse correlation between maternal placental DNA methylation levels and third-trimester HDL-C within the control group was found. These results were consistent with those of our previous studies [8–10] which highlighted the importance of lipid parameters related to genetic markers during the third trimester. Therefore, further studies are needed in order to determine how lipid profile can impair the placental epigenome.

The current literature has also proposed that DNA methylation patterns may be highly associated with specific genotypes, suggesting that the effect of genetic variants related to nutrients and metabolism may be exerted via epigenetic alterations and explaining the long-term interindividual variability in risk of obesity and diabetes [78, 79]. Our study showed that mothers’ *FTO* rs9939609 gene polymorphism presented an impact on birthweight. In fact, we found a higher birthweight expressed as percentiles in the offspring of women with rs9939609 *AA* genotype when compared with those with mothers carrying the *T* allele. Birthweight represents a predictor for the development, growth, and the afterwards adult period [80], and it is influenced by multiple factors and the interaction between them. In this context, the genetic susceptibility also seems an essential factor which may affect obesity-related phenotypes.

This study has some limitations. First of all, although 60 placental samples (total 120) can be considered a good sample size for DNA methylation, it should be noted that many DNA methylation patterns are tissue specific and cell specific (i.e. fat, liver, skeletal muscle, pancreatic islets). Therefore, it would be interesting to compare the *FTO* methylation profiles in placentas to DNA methylation levels in blood. Secondly, in order to provide an understanding of the link between metabolic programming and the increased incidence of metabolic diseases related to an altered intrauterine environment, future studies will be needed to investigate the possible correlation between obesity and DNA methylation levels, as well as the comparison of the different methylation patterns of GDM women treated with diet alone versus those treated with both diet and insulin.

In summary, the role of the maternal metabolism in regulating the placental epigenome still remains unclear. Although emerging evidence has demonstrated the fundamental role of *FTO* in the energy homeostasis and body composition, the effects of the epigenetic alterations of this gene as well as maternal metabolic disturbances are largely unexplored. It should be noted that all GDM participants were attentively supervised and proved to be fully compliant to the treatment, reaching their glycaemic targets: such thorough monitoring might have had a preventive effect on possible epigenetic modifications. A strength of this study is that, to our knowledge, this is the first research exploring the placental DNA methylation profile of *FTO* gene in GDM. In addition, this is the first attempt at the association between placental *FTO* DNA methylation and maternal metabolic characteristics (including unhealthy lifestyle and third-trimester lipid parameters). Therefore, our data offer some insights into the pathophysiology of obesity related to maternal habits, as well as to diet and environmental conditions during pregnancy. This perspective suggests the need for further insight into epigenetic modifications, so as to clarify the role of molecular mechanisms impacting on the foetus during pregnancy. So, we can say that in well-controlled GDM-affected women, placental epigenetic effects are related to the maternal smoking and TGs. Interestingly, an association between maternal *FTO* rs9939609 and neonatal birthweight was detected. In the awareness that the pathophysiology of obesity is very complex and in the recognition of the countless factors involved in long-term metabolic health of offspring of the GDM mothers, the present study adds a piece to this strenuous and intricate mosaic.

## Supplementary Information

Below is the link to the electronic supplementary material.

Supplementary file1 (DOCX 27 kb)

## Data Availability

The data underlying this article will be shared on reasonable request to the corresponding author.
