# Supplementary material for: Fat mass and obesity-associated (FTO) gene epigenetic modifications in gestational diabetes: new insights and possible pathophysiological connections
Source: Acta Diabetol. 2021 Mar 20;58(8):997–1007. doi: 10.1007/s00592-020-01668-5 (PMC8272710; doi:10.1007/s00592-020-01668-5)
Supplement: Supplementary file 1 — Supplementary file1 (DOCX 27 kb) [file 592_2020_1668_MOESM1_ESM.docx]

**Supplementary Table 1 – Tobit models predicting the DNA methylation percentages on the maternal side**

|  | CpG-1 | | CpG-2 | | CpG-3 | | CpG-4 | | Average | |
| --- | --- | --- | --- | --- | --- | --- | --- | --- | --- | --- |
|  | Coeff. (95% CI) | *p-value* | Coeff. (95% CI) | *p-value* | Coeff. (95% CI) | *p-value* | Coeff. (95% CI) | *p-value* | Coeff. (95% CI) | *p-value* |
| GDM vs. Control | 4.70 (-2.83; 12.24) | *0.221* | -0.39 (-3.11; 2.32) | *0.776* | 2.44 (-5.61; 10.43) | *0.552* | -0.25 (-8.67; 8.17) | *0.954* | 0.42 (-1.90; 2.73) | *0.725* |
| BMI pre | 0.52 (-0.06; 1.10) | *0.081* | -0.02 (-0.26; 0.23) | *0.890* | 0.33 (-0.35; 1.01) | *0.374* | -0.20 (-1.12; 0.72) | *0.669* | 0.10 (-0.08; 0.29) | *0.280* |
| BMI post | 0.46 (-0.04; 0.97) | *0.073* | -0.02 (-0.25; 0.21) | *0.884* | 0.32 (-0.29; 0.94) | *0.304* | -0.36 (-1.53; 0.81) | *0.550* | 0.12 (-0.06; 0.30) | *0.187* |
| Δ weight | 0.42 (-0.35; 1.190) | *0.283* | -0.05 (-0.37; 0.26) | *0.746* | 0.16 (-0.78; 1.09) | *0.742* | -0.01(-1.13; 1.10) | *0.981* | 0.12 (-0.14; 0.39) | *0.362* |
| PAS | 0.25 (-0.10; 0.61) | *0.166* | -0.01 (-0.12; 0.10) | *0.913* | 0.02 (-0.34; 0.38) | *0.902* | -0.07 (-0.41; 0.28) | *0.706* | -0.01 (-0.11; 0.09) | *0.873* |
| PAD | 0.17 (-0.20; 0.54) | *0.377* | 0.01 (-0.13; 0.14) | *0.930* | 0.22 (-0.25; 0.69) | *0.369* | -0.12 (-0.54; 0.30) | *0.576* | -0.01 (-0.14; 0.11) | *0.859* |
| Triglycerides | 0.04 (-0.03; 0.10) | *0.318* | 0.01 (-0.02; 0.03) | *0.648* | 0.04 (-0.06; 0.13) | *0.478* | 0.00 (-0.08; 0.07) | *0.915* | 0.00 (-0.02; 0.03) | *0.831* |
| HDL-c | -0.02 (-0.25; 0.21) | *0.849* | 0.03 (-0.07; 0.13) | *0.611* | -0.24 (-0.60; 0.12) | *0.197* | 0.14 (-0.15; 0.43) | *0.342* | -0.05 (-0.15; 0.04) | *0.262* |
| Smoker vs. non-smoker | -0.09 (-9.88; 9.70) | *0.986* | -1.26 (-4.45;1.94) | *0.441* | 6.50 (-2.33; 15.33) | *0.149* | -8.45 (-17.83; 0.94) | *0.997* | 0.75 (-1.64; 3.13) | *0.540* |
| Percentile weight | -0.07 (-0.28; 0.13) | *0.478* | -0.03 (-0.10; 0.03) | *0.277* | -0.09 (-0.30; 0.11) | *0.373* | -0.02 (-0.33; 0.29) | *0.892* | -0.05 (-0.12; 0.02) | *0.139* |

**Supplementary Table 2 – Tobit models predicting the DNA methylation percentages on the foetal side**

|  | CpG-1 | | CpG-2 | | CpG-3 | | CpG-4 | | Average | |
| --- | --- | --- | --- | --- | --- | --- | --- | --- | --- | --- |
|  | Coeff. (95% CI) | *p-value* | Coeff. (95% CI) | *p-value* | Coeff. (95% CI) | *p-value* | Coeff. (95% CI) | *p-value* | Coeff. (95% CI) | *p-value* |
| GDM vs. Control | 3.82 (-5.3; 12.9) | *0.411* | 0.80 (-2.03; 3.63) | *0.582* | 5.70 (-2.09; 13.50) | *0.151* | 2.82 (-7.45; 13.09) | *0.590* | 0.58 (-1.73; 2.88) | *0.623* |
| BMI pre | 0.38 (-0.36; 1.12) | *0.316* | 0.12 (-0.12; 0.36) | *0.311* | 0.26 (-0.39; 0.92) | *0.426* | -0.41 (-1.77; 0.95) | *0.554* | 0.05 (-0.15; 0.25) | *0.608* |
| BMI post | 0.28 (-0.51; 1.08) | *0.483* | 0.09 (-0.16; 0.34) | *0.473* | 0.04 (-0.63; 0.70) | *0.911* | -0.30 (-1.51; 0.91) | *0.625* | 0.02 (-0.17; 0.22) | *0.816* |
| Δ weight | -0.23 (-1.56; 1.11) | *0.738* | -0.07 (-0.44; 0.30) | *0.719* | -0.60 (-1.71; 0.51) | *0.292* | 0.03 (-1.14; 1.20) | *0.962* | -0.09 (-0.37; 0.19) | *0.522* |
| PAS | 0.18 (-0.30; 0.65) | *0.465* | 0.03 (-0.11; 0.16) | *0.689* | 0.34 (-0.05; 0.73) | *0.092* | -0.01 (-0.45; 0.43) | *0.962* | 0.08 (-0.04; 0.21) | *0.183* |
| PAD | 0.32 (-0.30; 0.94) | *0.313* | 0.03 (-0.13; 0.20) | *0.688* | 0.33 (-0.14; 0.81) | *0.167* | 0.03 (-0.49; 0.56) | *0.900* | 0.11 (-0.04; 0.27) | *0.152* |
| Triglycerides | 0.03 (-0.06; 0.11) | *0.507* | 0.03 (-0.01; 0.69) | *0.070* | 0.07 (-0.05; 0.19) | *0.228* | -0.03 (-0.15; 0.10) | *0.697* | 0.02 (-0.01; 0.05) | *0.122* |
| HDL-c | -0.11 (-0.37; 0.15) | *0.417* | 0.01 (-0.12; 0.12) | *0.937* | -0.16 (-0.53; 0.21) | *0.396* | -0.04 (-0.33; 0.24) | *0.760* | -0.01 (-0.11; 0.09) | *0.913* |
| Smoker vs. no smoker | -3.97 (-15.36;7.43) | *0.495* | 0.61 (-2.21; 3.44) | *0.669* | -0.44 (-8.54; 7.66) | *0.915* | -0.77 (-11.08; 9.54) | *0.884* | 0.28 (-2.19; 2.75) | *0.822* |
| Weight percentile | 0.01 (-0.18; 0.17) | *0.975* | 0.04 (-0.03; 0.11) | *0.231* | -0.05 (-0.21; 0.12) | *0.575* | -0.05 (-0.24; 0.15) | *0.623* | 0.01 (-0.05; 0.06) | *0.843* |

**Supplementary Table3. Percentage of methylation among women with at least 1% DNA methylation levels on the maternal side of placenta at the CpGs**

|  | OGTT at 16-18 wks | OGTT at 24-28 wks | p-value |
| --- | --- | --- | --- |
| *CpG-1* | 3.0 (1.5-4.0) | 9.5 (8.3-10.8) | 0.200 |
| *CpG-2* | 2.5 (2.3-2.8) | 2.5 (1.3-3.0) | 0.999 |
| *CpG-3* | 8.0 (4.0-8.0) | 4.0 (3.3-7.0) | 0.611 |
| *CpG-4* | - | 5.0 (4.0-6.0) | - |
| Average | 2.5 (1.8-3.3) | 1.0 (1.0-2.5) | 0.992 |

**Supplementary Table4. Percentage of methylation among women with at least 1% DNA methylation levels on the foetal side of placenta at the CpGs**

|  | OGTT at 16-18 wks | OGTT at 24-28 wks | p-value |
| --- | --- | --- | --- |
| *CpG-1* | 6.0 (6.0-6.0) | 9.0 (5.5-9.5) | 0.995 |
| *CpG-2* | 5.5 (5.3-5.8) | 3.0 (2.0-6.0) | 0.474 |
| *CpG-3* | 7.0 (6.0-8.0) | 3.0 (2.0-10.5) | 0.461 |
| *CpG-4* | - | 5.5 (5.3-5.8) | - |
| Average | 3.5 (2.8-4.3) | 1.0 (1.0-1.0) | 0.788 |
